# Nutrient retention after crop harvest in a typic hapludults amended with biochar types under no-tillage system

**DOI:** 10.1038/s41598-024-55430-w

**Published:** 2024-03-01

**Authors:** Qamar Sarfaraz, Gerson Laerson Drescher, Mohsin Zafar, Muhammad Nadeem Shah, Fengliang Zhao, Subhan Danish, Abd El-Zaher M. A. Mustafa, Mohamed S. Elshikh, Leandro Souza da Silva

**Affiliations:** 1 Department of Soil Science, Lasbela University of Agriculture, Water and Marine Sciences, Lasbela, Uthal Balochistan, Pakistan; 2 Federal University of Santa Maria, 1000 Roraima Ave, Santa Maria, RS 97105-900 Brazil; 3 Department of Environmental Sciences, Mirpur University of Science and Technology, Mirpur, AJK Pakistan; 4 University of Arkansas, Fayetteville, USA; 5 Department of Agriculture, Government College University Lahore, Lahore, Punjab Pakistan; 6 North Florida Research and Education Center, University of Florida, 155 Research Road, Quincy, FL USA; 7 Environment and Plant Protection Research Institute, Chinese Academy of Tropical Agricultural Science, Haikou, China; 8 Department of Soil Science, Faculty of Agricultural Sciences and Technology, Bahauddin Zakariya University, Multan, Punjab Pakistan; 9 Department of Botany and Microbiology, College of Science, King Saud University, P.O. 2455, 11451 Riyadh, Saudi Arabia

**Keywords:** Biochar, Stratification, pH, Exchangeable Al, Primary nutrients, Environmental sciences, Environmental chemistry, Abiotic

## Abstract

The utilization of biochar’s as soil amendments for enhancing nutrient retention in subsoils present potential limitations. To address this issue, we conducted a greenhouse experiment to assess the effects of various biochar’s derived from animal manures (swine manure, poultry litter, cattle manure) and plant residues (rice straw, soybean straw, corn straw) when applied to surface of an acidic soil. Our study focused on wheat crops under a no-tillage system, with a subsequent evaluation of the residual impacts on soybeans. The experimental design involved the application of biochar’s at different rates i.e. 10 and 20 Mg ha^−1^, followed by the assessment of their influence on NPK levels, pH, and exchangeable Al in stratified soil layers (0–5, 5–10, 10–15, and 15–25 cm). Furthermore, we investigated the interplay between biochar doses and the application of nitrogen (N) in the top 5 cm of soil, specifically examining ${\text{NO}}_{{3}}^{ - }$, ${\text{NH}}_{{4}}^{ + }$, P and K levels. Our findings revealed that in the top 5 cm of soil, biochar doses and N application significantly affected ${\text{NO}}_{{3}}^{ - }$, ${\text{NH}}_{{4}}^{ + }$, P and K concentrations. However, in deeper soil layers, no significant differences were observed among biochar doses with or without N application. Interestingly, K levels were impacted throughout all soil depths, regardless of the presence or absence of N application. Moreover, biochar application up to a 5 cm depth induced favorable changes in soil pH and reduced exchangeable Al. In contrast, deeper layers experienced a decrease in soil pH and an increase in exchangeable Al following biochar treatment. In conclusion, our study demonstrates that biochar’s can effectively retain NPK nutrients, enhance soil pH, and decrease exchangeable Al, independent of the type and dosage of application under a no-tillage system. Nonetheless, the efficacy of biochar amendments may vary with soil depth and type of nutrient, warranting careful consideration for maximizing their benefits in sustainable agricultural practices.

## Introduction

Biochar is a solid product derived from the carbonization or pyrolysis of biomass, such as agricultural residues, wood waste, or organic matter^1–4^. This process involves heating the biomass in a low-oxygen environment, preventing complete combustion, and resulting in the formation of a stable, carbon-rich material^5–8^. The production and application of biochar’s to soil instigate fundamental changes in soil nutrient cycling, leading to enhanced soil fertility and increased crop productivity^9–15^. Particularly in acidic, infertile soils with low organic matter content, biochar application yields positive responses. In acidic soils, the functional groups on biochar reduce aluminum phytotoxicity by forming surface complexes with aluminum cations^16^. As biochar application rate increased, significant increases were observed in exchangeable base cations and decreases in exchangeable acidity and Al saturation, but no additional changes in soil pH. The elimination of exchangeable acidity and the strong buffering capacity of biochar may be partially responsible for the lack of change in soil pH at the higher biochar application rate, inhibiting a further liming effect^17^. However, the outcomes of this application in such soils can exhibit variability^18,19^. The nitrification process is regulated by several factors, including soil pH, temperature, soil moisture, nitrogen (N) supplying substrate, soil microbes, and soil types. These elements play crucial roles in shaping the rate and efficiency of nitrification in the soil ecosystem^20^, as the nitrification process is major factor in N cycle in soil as well as nutrient use efficiency^21–23^. Understanding the nitrification process and its environmental implications for various soil types is crucial for enhancing soil fertility and promoting environmental protection. It is essential to comprehend soil processes affected by factors such as Low pH, high Al, and low CEC, which significantly limit crop growth. The common practice of using liming on acid soils to elevate pH and boost crop yields requires careful consideration^24^. Prolonged and excessive liming can lead to soil compaction, disrupt the balance of Ca, K, and Mg in the soil, ultimately resulting in reduced crop productivity^25^. A number of studies have been reporting to understand the nitrification and acidification in forests and temperate soil^26^. The attention towards the potential advantages of no-tillage has increased, particularly concerning carbon sequestration, CO_2_ emissions mitigation, and improvement of soil quality^27^. The understanding of ${\text{NH}}_{{4}}^{ + }$ application effects in tropical and subtropical regions under a no-tillage system is currently limited, with scant information available^28–30^. Considering the significance of managing acidic soils and improving soil fertility to enhance agricultural production, we chose to utilize biochar’s derived from animal manures and plant residues.

The biochars, mostly negatively charged material^31^ and being high surface charge can enhance the nutrient retention and use in soil^32^ having great potential for improving soil fertility^33^. Biochar application to soils can magnificently hold onto the nutrients that are required plants. However, the relationship between nutrient retention and loss pathways is still not obvious. The present study, we designed to find out the retention of nutrients C, N (${\text{NO}}_{{3}}^{ - }$, ${\text{NH}}_{{4}}^{ + }$), P, K and micronutrients through the application of animal manures and plant residues derived biochars under greenhouse conditions. Our objective was to raise soil pH, reduce soil acidity, and boost nutrients retention in soil by using biochars derived from animal manures and plant residues. This study focused on evaluating nutrient retention in the soil after two consecutive crops of wheat and soybean, comparing plots with and without ${\text{NH}}_{{4}}^{ + }$ fertilizer application. We hypothesized that the biochar’s from various animal manures and plant residues would enhance soil nutrient retention capacity and increase soil pH in different soil layers. Additionally, we expected that the biochar’s would slow down the nitrification process, thereby promoting the retention of nitrogen in the ${\text{NH}}_{{4}}^{ + }$ form.

## Material and methods

### Soil collection

The soil collection site was selected on basis of no-till areas, according to the data available from the Department of Soil Science of the Federal University of Santa Maria (29° 43′ 14.2″ S 53° 42′ 15.0″ W). The vegetative cover and grasses were removed manually prior to collecting the soil. The un-disturbed soil was collected in polyvinyl pipes (PVC) (0.29 m height × 0.20 m diameter) up to 25 cm were collected for experiments under no-tillage system for pre-sowing analysis and for experimental use. Prior to installing experiments, the soil was analyzed for pH (4.8 (1:2.5 w/v)), total C (1.2%), N (0.8%), P (4.8 mg kg^−1^), K (28 mg kg^−1^), Ca (15.5 cmol_c_ dm^−3^), Mg (9.3 cmol_c_ dm^−3^) and Al (16.89 cmol_c_ dm^−3^). The collected soil having sandy loam texture of the soil (61.71% sand, 25.72% silt, 12.56% clay) was classified as typic hapludults (USDA Soil Taxonomy).

### Biochar preparation and analysis

To prepare the biochars, data was collected to find out the quantity and type of feedstock available and decided to collect the materials available easily and are even having any kind of difficulty in their dispose-off. For biochar’s preparation, all feedstocks were collected from the experimental areas of the Federal University of Santa Maria—RS (29° 43′ 14.4″ S 53° 43′ 31.2″W) while corn straw was collected from a nearby city Paraíso do Sul—RS (29° 35′ 10.3″ S 53° 07′ 26.3″ W). Biochar’s, swine manure biochar (SMB), poultry litter biochar (PLB), cattle manure biochar (CMB), rice straw biochar (RSB), soybean straw biochar (SSB) and corn straw biochar (CSB) were prepared at 450 °C for 1 h in muffle furnace with an increase in temperature 10 °C min^−1^. All the biochar’s were analyzed for pH, electrical conductivity (EC) total carbon (C: Thermo Scientific, Flash EA 1112, Milan, Italy), total nitrogen (N: Thermo Scientific, Flash EA 1112, Milan, Italy), phosphorus (P: Murphy & Riley, 1962), potassium (K: Tedesco et al. 1995), calcium (Ca: Tedesco et al. 1995), magnesium and (Mg: Tedesco et al. 1995).

### Experimental setup and treatment plan

A greenhouse experiment was conducted to evaluate the influence of different biochar types on wheat under no-tillage system with biochar application rate at 0 (0 g column^−1^), 10 (33.5 g column^−1^) and 20 Mg ha^−1^ (67 g column^−1^) with three replicates and their subsequent effect on soybean under complete randomized design (CRD) with three factors i.e. biochar types, biochar dose, nitrogen levels (6 × 2 × 2) with two controls (control 1: No biochar, no nitrogen and control 2: no biochar, recommended nitrogen). Recommended doses of nitrogen (110 kg ha^−1^ ~ 1.6 g ammonium sulfate column^−1^), P_2_O_5_ (170 kg ha^−1^ ~ 1.3 g triple superphosphate column^−1^) and 120 kg K_2_O ha^−1^ (~ 0.65 g potassium chloride column^−1^) were also recommended along with biochar treatments.

As the biochars derived from plant residues had a huge volume, all the biochars were mixed up to 3 cm to have a good contact between soil and biochar to ensure seed placement in good contact with soil biochar mixture. Eight wheat seeds (Sinuelo variety) were sown into each PVC column, after germination thinning was done and four healthy seedlings were left for growth up to 93 days. After wheat harvest, three out of six soybean (5958 RSF IPRO variety) were left for 66 days with basal dose of 90 kg P_2_O_5_ ha^−1^ (0.69 g triple superphosphate column^−1^) and 120 kg K_2_O ha^−1^ (0.65 g potassium chloride column^−1^), but no nitrogen was added to PVC columns and after 66 days soybean aerial part was of soybean was collected for further analysis. The PVC columns were irrigated on daily basis depending upon the visual soil conditions due to the sandy loam texture of soil, to fulfill the water requirements of both crops respectively.

### Soil stratification

After the soybean harvest soil columns were cut into two halves vertically and stratified soil samples were as 0–5, 5–10, 10–15 and 15–25 cm to evaluate the influence of different biochar’s on nutrient retention in topsoil as well as subsoil. The stratified soil samples were then air-dried, ground and passed through 2 mm sieve, then were analyzed for ${\text{NH}}_{{4}}^{ + }$, ${\text{NO}}_{{3}}^{ - }$, P, K, Ca, Mg and Al in different soil layers through recommended procedures used in Soil Science Lab at Federal University of Santa Maria.

### Statistical analysis

**Figure 1:**
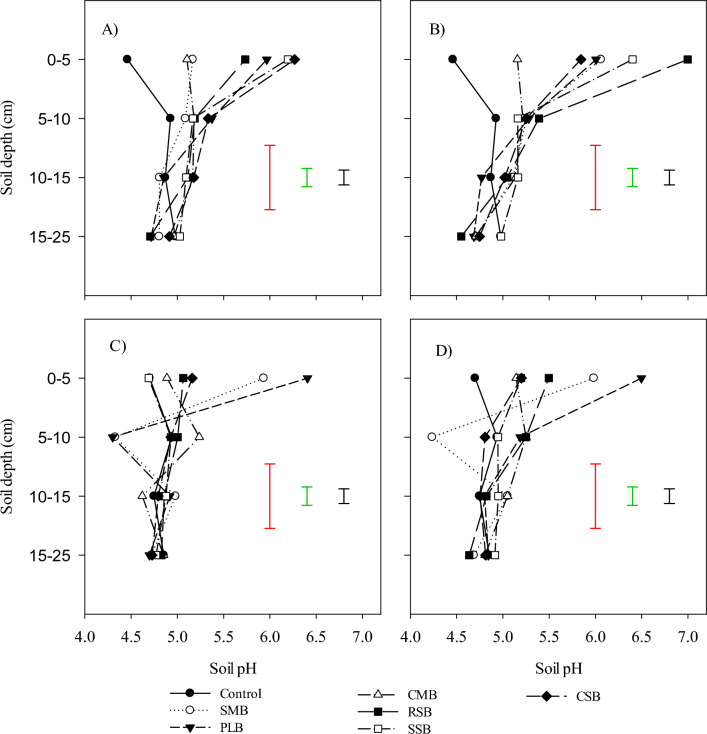
pH change in different soil layers with surface application of swine manure biochar (SMB), poultry litter biochar (PLB), Cattle manure biochar (CMB), rice straw biochar (RSB), soybean straw biochar (SSB) and corn straw biochar (CSB), (**A**) 10 Mg ha^−1^, (**B**) 20 Mg ha^−1^, (**C**) 10 Mg ha^−1^ with N and (**D**) 20 Mg ha^−1^ with N.

**Figure 2:**
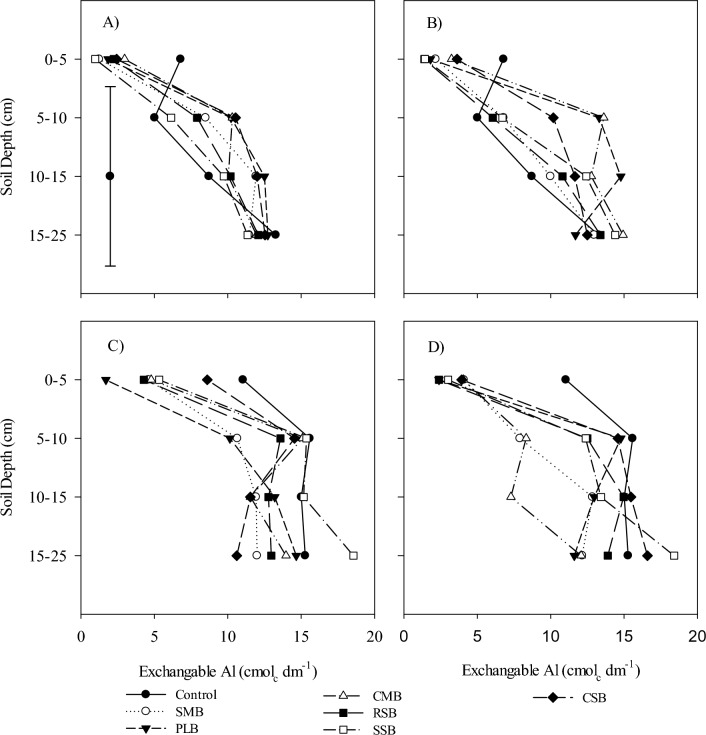
Soil exchangeable Al in different soil layers with surface application of swine manure biochar (SMB), poultry litter biochar (PLB), Cattle manure biochar (CMB), rice straw biochar (RSB), soybean straw biochar (SSB) and corn straw biochar (CSB), (**A**) 10 Mg ha^−1^, (**B**) 20 Mg ha^−1^, (**C**) 10 Mg ha^−1^ with N and (**D**) 20 Mg ha^−1^ with N.

Standard statistical analysis was performed on collected data^34^. Analysis of variance was conducted to check the significance of treatments and to compare means of the treatment with controls and with other treatment using software R using linear model (version 3.5) with compatible services by R-studio (version 1.1461). The mean comparison was done using Tukey Multiple comparison test at p < 0.05 using the “emmeans” package. The Figs. 1 and 2 were drawn through SigmaPlot 12.3 version.

### Ethics approval and consent to participate

We all declare that manuscript reporting studies do not involve any human participants, human data, or human tissue. So, it is not applicable. Our experiment follows the with relevant institutional, national, and international guidelines and legislation.

## Results and discussions

### Primary nutrients concentration

It has been recognized that biochars can adsorb both ${\text{NO}}_{{3}}^{ - }$ and ${\text{NH}}_{{4}}^{ + }$ nitrogen because of their large surface areas and presence of a range of different functional groups, consequently increasing the soil fertility and crop production. On biochar’s surface both acidic and basic sites can be found which can affect the adsorption of cations as well anions^35^. From the results (Table 1) in layer 0–5 cm, it can be seen that retention of ${\text{NO}}_{{3}}^{ - }$ is influenced by the different biochar types in both levels of application. Maximum ${\text{NO}}_{{3}}^{ - }$ (25.0 mg kg^−1^) was adsorbed in treatment with CSB. The increase in dose of biochars increases ${\text{NO}}_{{3}}^{ - }$ retention in soil. Minimum ${\text{NO}}_{{3}}^{ - }$ was found in PLB (3.0 mg kg^−1^) and control (5.5 mg kg^−1^) treatment respectively. The addition of N fertilizer had no significant effect on ${\text{NO}}_{{3}}^{ - }$ retention in top 5 cm soil while an increase in ${\text{NO}}_{{3}}^{ - }$ was observed when N was applied as ${\text{NH}}_{{4}}^{ + }$ form. The N application to soil decreased the ${\text{NO}}_{{3}}^{ - }$ in columns with CMB, SSB and CSB both in 10 as well as 20 Mg ha^−1^. Data on soil layer 5–10 cm (Table 2) shows that increase in depth of soil decreased the ${\text{NO}}_{{3}}^{ - }$ retention in soil both in different biochar types as well as doses of biochar’s. There was no effect was noted among different biochar types, even with different doses of biochar’s i.e., 10 and 20 Mg ha^−1^. The N application to crops also didn’t affect the ${\text{NO}}_{{3}}^{ - }$ in soil after harvest. Maximum ${\text{NO}}_{{3}}^{ - }$ (8.0 mg kg^−1^) was observed in soil column amended with SMB at 20 Mg ha^−1^ while Minimum (1.1 mg kg^−1^) was observed in soil column treated with SSB at 20 Mg ha^−1^. No ${\text{NO}}_{{3}}^{ - }$ was found in deeper layers i.e., 10–15 and 15–10 cm (Tables 3 and 4) even an application of N fertilizer to an acidic soil had no effect on soil ${\text{NO}}_{{3}}^{ - }$ contents under no tillage conditions. Presence of ${\text{NO}}_{{3}}^{ - }$ in soil layer 0–5 cm confirms the nitrification process occurs in topsoil which was mixed with biochar (2.5–3 cm) based on the great volumes of the plant residues derived biochars. The application of alkaline biochar with high adsorptive capability adsorbs ${\text{NO}}_{{3}}^{ - }$ and ${\text{NH}}_{{4}}^{ + }$ and hence reduce N loss from soil^36^. Biochars being porous and high exchange nature material can adsorb more nutrients, enhancing the soil nitrogen contents^37,38^. A high amount of nitrogen is attached by biochars when they are applied in high rates in soil^39^. The ${\text{NH}}_{{4}}^{ + }$ contents in soil were not affected by different biochar types after crop harvest (Table 1), even increase in dose of biochar’s had no significant effect in ${\text{NH}}_{{4}}^{ + }$ retention in soil in topsoil layer (0–5 cm). A slight increase in ${\text{NH}}_{{4}}^{ + }$ was observed with increase in dose of each biochar. Ammonium content in soil was also influenced directly with application of ${\text{NH}}_{{4}}^{ + }$ fertilizer in soil in wheat crop under no tillage system. The N application increased the ${\text{NH}}_{{4}}^{ + }$ retention in soil while the dose of biochar had not a significant effect on ${\text{NH}}_{{4}}^{ + }$ retention in top 5 cm soil layer. Maximum ${\text{NH}}_{{4}}^{ + }$ (67.6 mg kg^−1^) was observed with application of CSB at 20 Mg ha^−1^ whereas minimum was observed in control treatment (control with N application). The most important biochar physical property to retain ${\text{NH}}_{{4}}^{ + }$ and NH_3_ is the surface area and pore structure. The NH_3_ also act as Lewis’s acid that could react with carboxyl groups pf biochar and produce ${\text{NH}}_{{4}}^{ + }$ or amide group^40^. However, NH_3_ being an alkaline gas, the acidic surface groups on biochar with low pH can protonate NH_3_ gas to ${\text{NH}}_{{4}}^{ + }$ ions thereby promoting their adsorption onto the cation exchange sites of biochar^41^ hence reducing the ${\text{NH}}_{{4}}^{ + }$ loss through NH_3_. In soil layer 5–10 cm decreased ${\text{NH}}_{{4}}^{ + }$ content as compared to top 5 cm soil, the decrease in ${\text{NH}}_{{4}}^{ + }$ concentration shows the weak influence of surface application of biochar’s derived from animal manures and plant residues. Resaee et al.^42^ noted that biochars with higher O/C ratio can have more ${\text{NH}}_{{4}}^{ + }$ adsorption as compared to biochars with less O/C, likewise Wang et al.^43^ found a direct relationship between functional groups and ${\text{NH}}_{{4}}^{ + }$ adsorption. An increase can be seen with increase in dose of biochar’s but there was not statistically (p < 0.05) significant difference found between the two doses of biochars. The application of N to soil also didn’t affect the ${\text{NH}}_{{4}}^{ + }$ in soil up to 10 cm depth. A decrease and slight increase can be observed in both doses of biochar’s for example, in soil column SMB had ${\text{NH}}_{{4}}^{ + }$ contents 15.7 mg kg^−1^ at 10 Mg ha^−1^ that decreased with 20 Mg ha^−1^ to 6.6 mg kg^−1^ while in case of CSB increased from 9.3 mg kg^−1^ to 12.2 mg kg^−1^ with increase in dose of biochar. In both control treatments (with N and without N) ${\text{NH}}_{{4}}^{ + }$ was almost same 15.5 mg kg^−1^ without N and 14.6 m kg^−1^ with N application. As compared to ${\text{NO}}_{{3}}^{ - }$, the ${\text{NH}}_{{4}}^{ + }$ was found continuously up to 25 cm layers collections (Tables 3 and 4), but with the increase in soil depth the concentration also remained gradually decreasing. There are number of studies showing that the addition of biochars lower the loss of ${\text{NO}}_{{3}}^{ - }$ through leaching and increase its concentrations in soil were for short period of time while long term experiments were still overlooked. According to Coa et al.^44^, the inclusion of biochar improved ${\text{NO}}_{{3}}^{ - }$ retention in the early phases of the experiment, while ${\text{NO}}_{{3}}^{ - }$ N loss by leaching increased in the later stages. Kameyama et al.^45^ reported adsorption of ${\text{NO}}_{{3}}^{ - }$ primarily caused by base functional groups rather than physical sorption thus biochar and NO_3_^−^adsorption relationship is weak. On the other hand, the ${\text{NO}}_{{3}}^{ - }$ adsorption may be attributed to electrostatic interactions and ion exchange phenomena^46^. The increase in dose of biochar’s had also a little influence in ${\text{NH}}_{{4}}^{ + }$ contents whereas there was not a significant between doses of biochar’s, even in case of SSB and CSB the ${\text{NH}}_{{4}}^{ + }$ content decreased 78 and 38% respectively with increase in dose of biochar (Table 3). With increase in soil depth the ${\text{NO}}_{{3}}^{ - }$ contents decreased and in final 2 layers (10–15 and 15–25 cm) no ${\text{NO}}_{{3}}^{ - }$ was noted that can be directly attributed to the no tillage soil conditions that we couldn’t mix the soil and biochar at grater depths. The available P remained changing with increase in depth, in top 0–5 cm layer P was influenced with biochar types as well as the increase in dose of biochar’s under no tillage system. Highest P (177.9 mg kg^−1^) was found in soil column treated with CMB at 10 Mg ha^−1^ while minimum (33.7 mg kg^−1^) was observed in control (no biochar, no N). The addition of N fertilizer enhanced the P retention in soil in all treatments with 20 Mg ha^−1^ except the soil treated with SMB where the addition of N fertilizer decreased the P content in soil i.e., 247 mg kg^−1^ without N and 206.6 mg kg^−1^ with the addition of N, while the P contents remained non-significant with biochar’s dose at 10 Mg ha^−1^ with N application together. In control treatments addition of N also increased the P retention in soil. With increase in depth, decrease in available P (Table 2) was observed but among different biochar types, no difference was observed when applied at 10 and 20 Mg ha^−1^ without N fertilizer while a huge increase was noted in column treated with SMB at 20 Mg ha^−1^ as compared to 10 Mg ha^−1^. The addition of N fertilizer also had not a significant impact on available P contents between 5 and 10 cm depth. In control treatments, no difference was found with and without application of mineral N fertilizer. As compared to topsoil layers 0–5 and 5–10 cm, the available P in the subsoil layers (10–15 and 15–25 cm) was remained uninfluenced with different biochar types, doses of biochar’s as well as in combination with mineral N fertilizer (Tables 3 and 4), while a minute different among treatments and doses can be noted. In the acidic soils, the P sorption is higher than the neutral or alkaline soil because of its low pH and Fe, Al and Mn oxides are dominant at low pH and fix P and reduce its availability^47^. Addition of biochar’s in low pH soil can decrease the soil pH and increase available P in soil solution by the increase in negative charged surfaces and pH may be increased by proton consumption reaction and hence forming hydro-oxides of Al and Fe. The biochar types, doses of biochar’s as well as combination of N strongly affected the available K in soil after the crop harvest. The available K ranged from 15 to 248 mg kg^−1^ affected by control (no biochar, no N) and RSB respectively without an application of N fertilizer (Table 1). The application of N decreased the k retention significantly in both 10 and 20 Mg ha^−1^. With increase in soil depth the available K concentration decreased while the influence of different biochar’s on K remained significant among different biochar types, doses, and combination of N. in sublayer 5–10 cm highest K (267.7 mg kg^−1^) was observed in soil column treated with RSM at 20 Mg ha^−1^ while minimum (14.3 mg kg^−1^) was observed in control treatment (no biochar, no N) (Table 2). From the data (Tables 3 and 4) similar behavior has been observed that with increase in biochar dose the K content increases while the addition of mineral N fertilizer decreases the K contents in soil.

**Table 1:** Nutrients concentration in 0–5 cm after crop harvest with application of swine manure biochar (SMB), poultry litter biochar (PLB), Cattle manure biochar (CMB), rice straw biochar (RSB), soybean straw biochar (SSB) and corn straw biochar (CSB) under no-tillage system.

(mg kg^−1^)	Control	10 Mg ha^−1^						20 Mg ha^−1^					
		SMB	PLB	CMB	RSB	SSB	CSB	SMB	PLB	CMB	RSB	SSB	CSB
Without N													
NO_3_	5.5 cA	15.1bAα	3.0 cBα	20.4aAß	15.8 bAα	21.5 aAα	25.0 aAß	14.4 cBα	3.6 dBα	33.0 bBα	4.8 dBß	29.5 bBα	45.0 aBα
NH_4_	1.2aAß	15.8aAα	13.2aBα	50.0aAα	17.1aBα	45.5aAα	39.8aAα	37.1aAα	18.2aAα	20.3aAß	14.4aBα	27.8aAα	27.1aBα
P	33.7bB	130.7aAß	64.4bAß	177.9aAα	30.1cBα	33.2cBα	19.3cBα	247.9aAα	128.4bBα	24.4cBß	27.9cBα	38.9cBα	77.2bBα
K	15.3cA	69.3bAα	58.3bAß	59.0bAß	105.7aAß	42.0cAα	80.3bAα	75.3cAα	137.7bAα	88.7cAα	248.7aAα	38.3cAα	98.7cAα
With N													
NO_3_	5.9 cA	19.1 aAß	11.5 aBα	8.4 cBα	17.3 aAß	2.4 dBα	4.3 dBα	26.5 aAα	13.7 bAα	10.4 bAα	22.4 aAα	5.4 cAα	14.5 bAα
NH_4_	19.6aAα	33.5aAα	39.6aAα	33.8aAα	62.2aAα	35.9aAα	37.6aAß	23.6aAα	37.7aAα	34.2aAα	50.9aAα	36.0aAα	67.6aAα
P	117.8aA	114.9aAß	97.7aAß	135.2aAα	163.4aAα	173.4aAα	133.1aAß	206.6aAα	160.5aAα	186.4aAα	188.8aAα	197.3aAα	220.3aAα
K	18.7aA	42.7bBß	55.7aAß	31.0bBß	52.0aBß	22.0bBα	33.7bBα	69.3cAα	97.3aBα	58.3cBα	117.3aBα	29.0dAα	50.7cBα

Small letters: = biochar type; capital letters = biochar dose; Alpha beta = with and without nitrogen respectively. The LSD values are ${\text{NO}}_{{3}}^{ - }$ (6.65), ${\text{NH}}_{{4}}^{ + }$ (35.33), P (83.19), and K (27.12).

**Table 2:** Nutrients concentration in 5–10 cm after crop harvest with application of swine manure biochar (SMB), poultry litter biochar (PLB), Cattle manure biochar (CMB), rice straw biochar (RSB), soybean straw biochar (SSB) and corn straw biochar (CSB) under no-tillage system.

(mg kg^−1^)	Control	10 Mg ha^−1^						20 Mg ha^−1^					
		SMB	PLB	CMB	RSB	SSB	CSB	SMB	PLB	CMB	RSB	SSB	CSB
Without N													
NO_3_	7.8aAα	6.9aAα	1.1aAα	1.2aAα	6.7aAα	1.00aAα	2.5aAα	8.0aAα	1.0aAα	6.0aAα	5.4aAα	6.0aAα	5.8aAα
NH_4_	15.5aA	14.0aAα	15.0aAα	13.6aAα	15.0aAα	17.3aBα	9.2aAα	14.7aAα	15.6aAα	10.8aAα	13.2aAα	10.7aAα	15.4aAα
P	8.5bA	53.1aBß	17.1aAα	12.9aAα	16.3aAα	19.5aAα	36.6aAα	178aAα	22.1bAα	11.6bAα	14.5bAα	34.8bAα	18.1bAα
K	14.3cA	54.0bAß	79.7bAß	33.5cAα	114.7aAß	29.7cAß	60.7bBß	136.3aAα	133.3bAα	56.3cAα	267.7aAα	31.0cAα	122.3bAα
With N													
NO_3_	3.6aAα	1.6aAα	3.2aAα	7.1aAα	4.6aAα	1.2aAα	1.1aAα	6.7aAα	1.2aAα	1.8aAα	2.6aAα	1.1aAα	1.3aAα
NH_4_	14.6aA	15.7aAα	11.8aAα	9.6aAα	10.0aAα	9.4aAα	9.3aAα	6.6aBα	10.8aAα	12.9aAα	12.7aAα	8.7aAα	12.2aAα
P	9.2cA	108aAß	21.2bAα	7.3bAα	9.4bAα	36.1bAα	15.9bAα	163aAα	26.1bAα	13.0bAα	18.1bAα	19.0bAα	17.5bAα
K	15cA	32.3bAß	41.3bBß	11.3bAα	65.3aBß	13.3bAα	22.3bAß	73.0cBα	123.3bAα	32.0cAα	205.3aBα	11.3dAα	55.3cBα

Small letters: = biochar type; capital letters = biochar dose; Alpha beta = with and without nitrogen respectively. The LSD values are ${\text{NO}}_{{3}}^{ - }$ (8.12), ${\text{NH}}_{{4}}^{ + }$ (8.76), P (51.43), and K (39.60).

**Table 3:** Nutrients concentration in 10–15 cm after crop harvest with application of swine manure biochar (SMB), poultry litter biochar (PLB), Cattle manure biochar (CMB), rice straw biochar (RSB), soybean straw biochar (SSB) and corn straw biochar (CSB) under no-tillage system.

(mg kg^−1^)	Control	10 Mg ha^−1^						20 Mg ha^−1^					
		SMB	PLB	CMB	RSB	SSB	CSB	SMB	PLB	CMB	RSB	SSB	CSB
Without N													
NO_3_	0	0	0	0	0	0	0	0	0	0	0	0	0
NH_4_	9.2Aα	7.0bAα	2.4bAα	6.0bAα	2.4bAα	23.4aAα	12.7bAα	12.3aAα	6.1aAα	5.6aAα	8.2aAα	4.5aAα	7.8aAα
P	7.8 ns	10.8 ns	29.4 ns	21.3 ns	18.3 ns	11.4 ns	67.9 ns	35.7 ns	12.5 ns	8.6 ns	16.4 ns	48.5 ns	25.6 ns
K	0.7bA	21.7bAß	39.3bAß	15.0bAα	60.6aAß	19.0bAα	26.3bAß	68.3bAα	69.0bAα	28.3cAα	121.0aBα	26.7cAα	53.3bAα
With N													
NO_3_	0	0	0	0	0	0	0	0	0	0	0	0	0
NH_4_	13.2Aα	4.8aAα	11.7aAα	11.6aAα	4.2aAα	8.0aAα	13.1aAα	12.8aAα	13.4aAα	8.0aAα	20.24aAα	6.5aAα	13.2aAα
P	11.6 ns	19.8 ns	28.7 ns	6.9 ns	13.3 ns	14.2 ns	15.2 ns	21.9 ns	27.4 ns	30.5 ns	21.9 ns	66.9 ns	17.7 ns
K	11bA	17.3bAß	18.3bBß	12.3bAα	38.7aBß	12.0bAα	15.6bAα	42.7cBα	76.3bAα	17.7dAα	153.0aAα	9.3dBα	29.0dBα

Small letters: = biochar type; capital letters = biochar dose; Alpha beta = with and without nitrogen respectively. The LSD values are ${\text{NO}}_{{3}}^{ - }$ (2.22), ${\text{NH}}_{{4}}^{ + }$ (19.93), P (83.61), and K (23.92).

**Table 4:** Nutrients concentration in 15–25 cm after crop harvest with application of swine manure biochar (SMB), poultry litter biochar (PLB), Cattle manure biochar (CMB), rice straw biochar (RSB), soybean straw biochar (SSB) and corn straw biochar (CSB) under no-tillage system.

(mg kg^−1^)	Control	10 Mg ha^−1^						20 Mg ha^−1^					
		SMB	PLB	CMB	RSB	SSB	CSB	SMB	PLB	CMB	RSB	SSB	CSB
Without N													
NO_3_	0	0	0	0	0	0	0	0	0	0	0	0	0
NH_4_	7.2aA	11.7aAα	9.2aAα	10.2aAα	8.4aBα	7.9aBα	13.4aBα	18.4aBα	13.1aAα	9.9aAα	8.9aα	10.6aBα	14.76aAα
P	4.7aB	18.9aAα	6.4aAα	4.3aAα	4.7aAα	2.9aAα	3.6aAα	22.1aAα	8.0aAα	3.5aAα	3.4aAα	3.7aAα	4.3aAα
K	7.3bA	24.3bAα	20.7bAß	15.0bAα	39.7aAα	14.3bAα	27.3bAα	32.6bAα	49.0aAα	17.0cAα	39.0bAα	19.7cAα	23.3cAα
With N													
NO_3_	0	0	0	0	0	0	0	0	0	0	0	0	0
NH_4_	10.6aA	13.1cAα	8.0cAα	17.5cAß	38.5bAα	50.5aAα	52.3aAα	15.2cAα	10.8cAα	57.6aAα	30.9bα	34.3bAα	49.3aAα
P	18.9aA	16.6aAα	15.5aAα	14.0aAα	11.0aAα	7.8aAα	4.7aAα	21.8aAα	9.7aAα	10.2aAα	11.9aAα	22.4aAα	8.8aAα
K	7.7aA	14.7aAα	19.0aAα	19.3aAα	23.7aAß	13.3aAα	16.6aAα	27.0aAα	26.0aBα	22.0aAα	38.0aAα	12.7bAα	22.3aAα

Small letters: = biochar type; capital letters = biochar dose; Alpha beta = with and without nitrogen respectively. The LSD values are ${\text{NO}}_{{3}}^{ - }$ (0.00), ${\text{NH}}_{{4}}^{ + }$ (23.76), P (18.35), and K (17.89).

### Soil pH and Al alteration

Biochar pH ranges from 5.5 to 10.5, that depends on content and composition of the mineral fractions that may be different depending upon the feedstock and pyrolysis^48–52^ That’s why biochar can alter the ${\text{NH}}_{{4}}^{ + }$ and ${\text{NO}}_{{3}}^{ - }$ dynamic in soil system through their adsorptive properties and pH. Soil pH was greatly influenced by the addition of biochar’s alone, and along with ${\text{NH}}_{{4}}^{ + }$ fertilizer (Fig. 1A, B, C and D). The addition of biochar’s at 10 Mg ha^−1^ increased the soil pH sufficiently, highest soil pH was observed from the soil column treated with CSB, SSB, PLB and RSB as well in layer 0–5 cm. With increase in soil depth pH also decreased gradually even CSB, SSB and PLB decreased in layer 5–10 cm and remained decreased up to 15–25 cm (Fig. 1A). Increased dose of biochar’s also increased the soil pH drastically. Soil treated with RSB at 20 Mg ha^−1^ showed maximum soil pH, whereas SSB didn’t increase the soil pH with an increase in its dose (Fig. 1B). On the other hand, RSB decreased the soil pH in sublayer (15–25 cm) again up to an acidic level. Application of ammonium fertilizer also had an influence on soil pH in layer 0–5 cm, because in addition to NH_4_ fertilizer pH was increased up to a certain level (PLB, SMB), after that level then decreased quickly in 5–10 cm layer at 10 Mg ha^−1^ while PLB remained in slow decrease as compared to SMB at 20 Mg ha^−1^. In deeper soil layers there are not significant differences can be noted but the pH remained decreasing with increase in soil depth. The pH increase in surface layer can be related to the presence of biochar’s negatively charged phenolic, carboxyl and hydroxyl groups on surface of biochar which tend to bind H^+^ from soil solution by reducing soil H^+^ and hence increase in pH^53,54^. The pH increment increases the CEC by reducing the base cations leaching in competition H^+^ ions^55^. In our studies the biochar affected the only surface layer while underneath layers were not affected directly with addition of biochar even at 20 Mg ha^−1^. The addition of ${\text{NH}}_{{4}}^{ + }$ as fertilizer in soil decreases the soil pH whereas an increase occurs with application of biochar to an acid soil^56^ A huge gradient can be seen by addition of different biochar’s in soil under undisturbed soil (no tillage system). Minimum exchangeable Al was observed in soil layer 0–5 cm (Fig. 2A, B, C, D), that kept it increasing with increase in soil depth. Lowest exchangeable Al was observed in SSB at both 10 and 20 Mg ha^−1^. The addition of ammonium fertilizer didn’t influence the exchangeable Al content in an acidic soil under no tillage system while the influence of amendment was limited to a very shallow depth (5 cm), after that remained increasing and reached near to its original Al content in both 10 and 20 Mg ha^−1^. The addition of biochar’s increases the alkaline metals (Ca^2+^, Mg^2+^ and K^+^) oxides in acidic soil and hence soluble Al^3+^ reduces by an increase in pH^3,57–61^.

## Conclusion

Surface application of different biochar can have a limited impact on soil nutrients especially to an acidic soil. The biochar’s had a significant effect up to 5 cm soil depth by retaining ${\text{NO}}_{{3}}^{ - }$ while can hold higher quantities of ${\text{NH}}_{{4}}^{ + }$ up to more depths under no tillage system. Phosphorus can be adsorbed by biochar’s when applied at surface while in deeper layers biochar’s don’t influence the P retention in soil. Potassium is greatly influenced with surface application of biochar’s but decrease the K retention in soil with application of ${\text{NH}}_{{4}}^{ + }$ fertilizer together. Soil pH and exchangeable Al also can have a prodigious positive impact up to a certain depth with superficial application of biochar’s that may not have an impact in depth.

## Data Availability

All data generated or analyzed during this study are included in this published article.
